# Computational Strategy for Minimizing Mycotoxins in Cereal Crops: Assessment of the Biological Activity of Compounds Resulting from Virtual Screening

**DOI:** 10.3390/molecules27082582

**Published:** 2022-04-16

**Authors:** Vessela Atanasova, Emmanuel Bresso, Bernard Maigret, Natalia Florencio Martins, Florence Richard-Forget

**Affiliations:** 1 INRAE, UR 1264 Mycology and Food Safety (MycSA), F-33882 Villenave d’Ornon, France; vessela.atanasova@inrae.fr; 2 Université de Lorraine, CNRS, Inria, LORIA, F-54000 Nancy, France; emmanuel.bresso@loria.fr (E.B.); bernard.maigret@loria.fr (B.M.); 3 EMBRAPA Agroindustria Tropical, Fortaleza 60511-110, CE, Brazil; natalia.martins@embrapa.br

**Keywords:** *Fusarium graminearum*, *Fusarium culmorum*, type B trichothecenes, drug design, experimental validation

## Abstract

Cereal crops are frequently affected by toxigenic *Fusarium* species, among which the most common and worrying in Europe are *Fusarium graminearum* and *Fusarium culmorum*. These species are the causal agents of grain contamination with type B trichothecene (TCTB) mycotoxins. To help reduce the use of synthetic fungicides while guaranteeing low mycotoxin levels, there is an urgent need to develop new, efficient and environmentally-friendly plant protection solutions. Previously, *F. graminearum* proteins that could serve as putative targets to block the fungal spread and toxin production were identified and a virtual screening undertaken. Here, two selected compounds, M1 and M2, predicted, respectively, as the top compounds acting on the trichodiene synthase, a key enzyme of TCTB biosynthesis, and the 24-sterol-C-methyltransferase, a protein involved in ergosterol biosynthesis, were submitted for biological tests. Corroborating in silico predictions, M1 was shown to significantly inhibit TCTB yield by a panel of strains. Results were less obvious with M2 that induced only a slight reduction in fungal biomass. To go further, seven M1 analogs were assessed, which allowed evidencing of the physicochemical properties crucial for the anti-mycotoxin activity. Altogether, our results provide the first evidence of the promising potential of computational approaches to discover new anti-mycotoxin solutions

## 1. Introduction

Fusarium Head Blight (FHB) and Gibberella Ear Rot (GER) are two of the more devastating fungal diseases of small-grain cereals and maize, respectively [1]. In addition to direct losses related to the alteration in grain filling, FHB and GER pose potential health risks to domestic animals and humans due to the production of type B trichothecene mycotoxins (TCTB) by the associated pathogens [2,3,4]. *Fusarium graminearum* and *Fusarium culmorum*, the two main *Fusarium* species responsible for the FHB and GER diseases, are the main producers of TCTB that include deoxynivalenol (DON) and its acetylated forms, 3-acetyl-deoxynivalenol (3-ADON) and 15-acetyl-deoxynivalenol (15-ADON), and nivalenol (NIV) and its acetylated form 4-acetylnivalenol or fusarenone X (FX).

Due to its frequent occurrence in cereal crops and its recognized toxicity, DON is of major concern [5]. Maximum contamination levels acceptable for cereals and maize-based food were set by the European Commission in June 2005 (EC No. 856/2005) and revised in July 2007 (EC No. 1126/2007). Grains or derived products exceeding the established limits for DON, fixed to 1250 µg kg^−1^ for common wheat and 1750 µg kg^−1^ for durum wheat and maize, cannot be commercialized for human consumption. The toxicity of TCTB to humans and animals is well documented [6]. Symptoms can be highly diverse, depending on the animal species, the time of exposure and the levels of ingested mycotoxins. The toxic effects of TCTB include the alteration of immune functions, gastrointestinal disturbances (vomiting) and reduced ovarian function and reproductive disorders [7]. At the molecular level, DON induces ribosome and endoplasmic reticulum stress response and damage to mitochondria [6,8].

TCTB are heat-stable molecules and are not fully eliminated through the processes currently used in cereal-based food manufacture [9]. Thus, the best way to prevent the contamination would be to limit TCTB production at the field level during plant cultivation. Combined with good agricultural practices, the use of fungicides is a key factor in the integrated management strategies aimed at controlling mycotoxins [10]. However, their repeated use over decades has resulted in the worldwide emergence of fungal resistance [11]. New antifungals combining efficiency, specificity and low environmental impact are therefore needed, which implies identifying new fungal targets. Computational approaches that have so far been mainly and extensively used towards the development of novel drugs in the pharmaceutical industry could provide a powerful toolbox for the design of new fungicides [12]. Such a computational approach has been used by Martins et al. [13] to identify, from the *F. graminearum* PH-1 genome, interesting proteins that could serve as putative targets for blocking the fungal spread and the toxin production associated with FHB disease. Additional criteria used in the former search of protein targets included the expression during plant infection, cell localization in cytoplasm and accessibility to chemical compounds, exclusivity in fungi and absence in organisms such as insects, plants and humans. The present study will focus on two of the highlighted protein targets: the 24-sterol C-methyltransferase (ERG6) and the trichodiene synthase (TRI5). ERG6 is a non-essential enzyme involved in ergosterol biosynthesis, which has been recently reported as a promising target for new antifungal drugs [14]. TRI5 catalyzes the production of trichodiene, which is the first committed step in the TCTB biosynthetic pathway. A total of 10,240 molecules coming from the Life Chemicals (https://lifechemicals.com/ (accessed on 10 October 2019)) diversity libraries were docked to the two tridimensional target models of ERG6 and TRI5 by Martins et al. [13], leading to the selection of 15 putative inhibitors for each protein.

**Figure 1:**
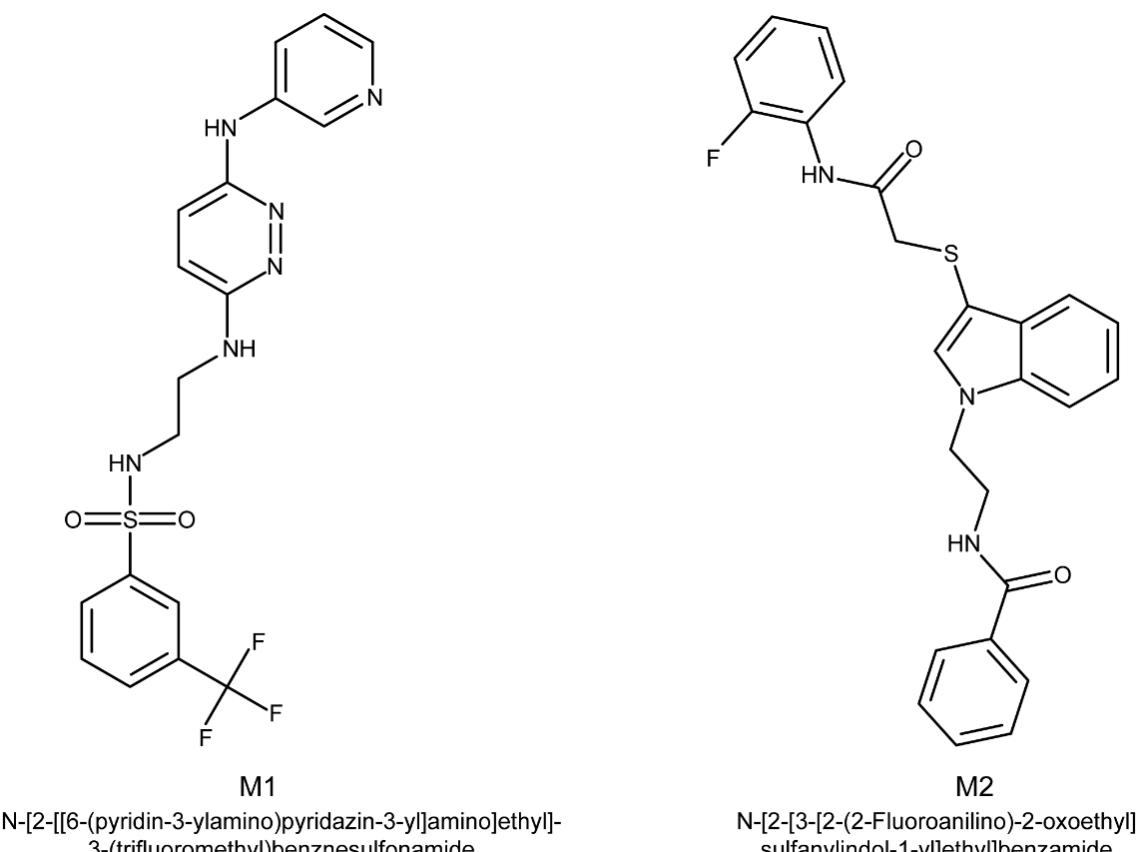
M1 and M2 chemical structures.

The present study aimed to check the validity of the previous in silico predictions using real world testing procedures. As a first proof of concept, we intended to measure the effects of some selected compounds on the growth and production of mycotoxins by *F. graminearum* and *F. culmorum* strains. The top compound of each docking campaign, namely Life Chemicals F5258-0045 (M1) for TRI5 and F0554-0055 (M2) for ERG6 (Figure 1), were considered. In a second step, to deepen the experimental results, our assays were extended to a set of analogs of M1 molecules, the selection of which within the Life Chemicals libraries was based on their Tanimoto coefficients [15].

## 2. Results

### 2.1. Impact of M1 and M2 on the Fungal Growth and Mycotoxin Accumulation by F. graminearum CBS 185.32

The effect of M1 at 50 and 150 µM and of M2 at 5 and 15 µM on fungal development and TCTB biosynthesis was evaluated (Figure 1). A slight but statistically significant inhibition of the fungal growth (lower than 5%) was observed in only one condition when culture media were supplemented with the M2 molecule at 15 µM (data not shown).

**Figure 2:**
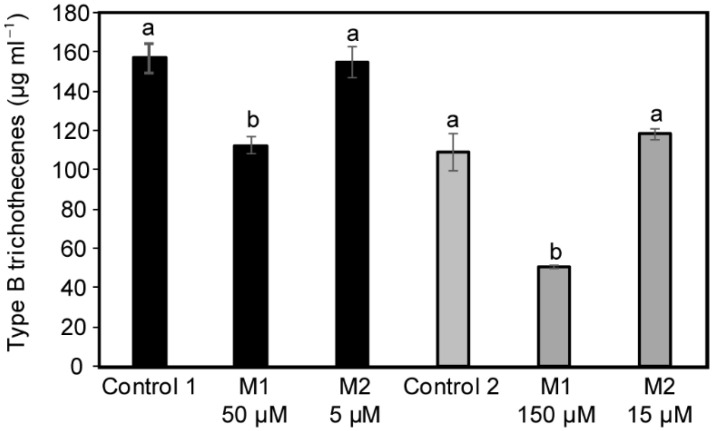
Effect of 50 µM (black) and 150 µM (gray) M1 and 5 µM (black) and 15 µM (gray) M2 on type B trichothecene accumulation by *Fusarium graminearum* CBS 185.32. Data are means ± standard deviation using three biological replicates. Different letters designate statistically significant differences between data from a same group gray or black (Kruskal–Wallis one-way analysis, with mean comparisons performed using Connover-Inman test, α ≤ 0.10). Control 1–DMSO 1%, Control 2–DMSO 3%.

With regard to the mycotoxin production, 15-ADON was the major TCTB produced by the studied strain in liquid media. The DON/15-ADON ratio was not significantly altered by the treatments, and the sum DON + 15-ADON was used to quantify the total TCTB produced (Figure 2).

In control conditions, the TCTB production reached 160 µg ml^−1^ and 108.7 µg ml^−1^ after 11 days of incubation, respectively. A significant inhibition of the mycotoxin biosynthesis, nearly 28% and 67%, was observed when culture media were supplemented with M1 at 50 and 150 µM, respectively. Supplementation with M2 at 5 µM and 15 µM did not affect the TCTB yield.

### 2.2. Effect of M1 on the Mycotoxin Production by Eight Fusarium Strains

Once the ability of the M1 compound to inhibit the production of TCTB was evidenced using one *Fusarium* strain, it was essential to check that this biological activity was confirmed using a panel of different strains. Thus, the effect of 150 µM M1 on fungal development and TCTB biosynthesis was investigated in liquid cultures using a set of seven additional TCTB-producing *Fusarium* strains that included the *F. graminearum* PH-1 strain, the genome of which was used for the selection of target proteins, and six strains of *F. culmorum* characterized by different chemotypes (Table 1). These *Fusarium* strains can be divided into three groups according to their ability to produce TCTB in liquid cultures (Table 1): low-producing (Fg PH-1, Fc 130, Fc 337, MCf 21, Fc 124), average-producing (Fc 319 and Fc 305) and high-producing (CBS 185.32) strains. 15-ADON, 3-ADON or FX were the major TCTB produced in liquid media by the studied strains, in accordance with their reported chemotype. The DON/15-ADON, DON/3-ADON and NIV/FX ratios were not significantly altered by the M1 treatments, and the sum DON + 15-ADON, DON + 3-ADON and NIV + FX was used to quantify the total of TCTB produced (Table 1).

Except for the Fc 305 strain, supplementation of the liquid broths with the M1 molecule induced a reduction in TCTB yield. For the Fc 319 and MCf 21 strains, a weak inhibition (lower than 20%) was observed, which was, however, not statistically significant when compared to the corresponding controls (Table 1). A high percentage inhibition was observed for two strains (PH-1 and Fc 124), with 100% and 52% of TCTB reduction after 11 days of culture, respectively. For three strains, CBS 185.32, Fc 130 and Fc 337, the decrease in TCTB accumulation was less drastic, ranging from 36% to 24%.

### 2.3. Search for M1 Analogs

Use of a Tanimoto analysis to screen the Life Chemical libraries resulted in the identification of 72 molecules presenting close chemical similarities with the M1 compound. Docking calculations, using the same docking procedure and the TRI5 tridimensional model that were used previously in our virtual screening campaign [13] were performed on these 72 molecules. A particular emphasis was put on TRI5 since M1 was the compound presenting the top docking score for this protein from the whole diversity set of chemical samples used in our previous virtual screening work.

**Figure 3:**
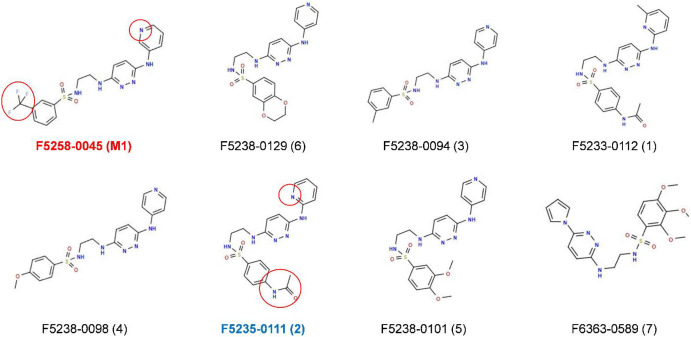
Chemical structures of the seven analogs versus F5228-0045 (M1) (in red). Comparison of M1 and analog 2 (in blue) showing their analogy and differences (in red circles). The number in parentheses corresponds to the analog as described in Table 2.

After the docking calculations, and considering additional filters such as lipophilicity predicted according to Tetko and Tanchuk [17] and toxicity predicted according to Banerjee et al. [18], only seven compounds were retained (see Table 2 and Figure 3) and tested for their biological activity towards *F. graminearum*. As reported in Table 2, all the seven compounds were lipophilic molecules. 

### 2.4. Impact of the Seven Additional Compounds on the Mycotoxin Production by F. graminearum Strain CBS 185.32

**Figure 4:**
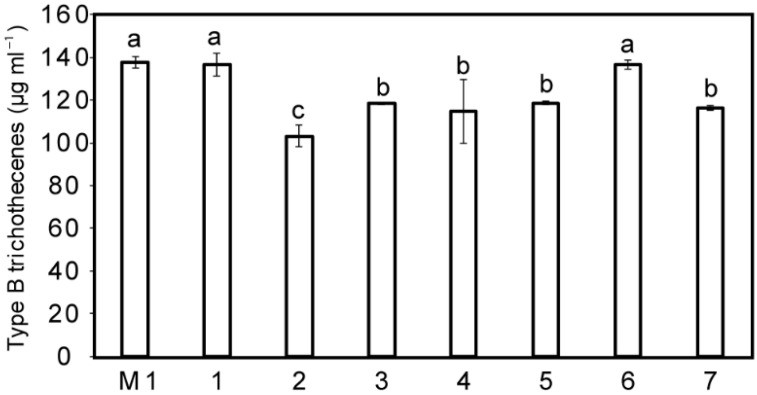
Effect of seven M1 analogs and M1 at 150 µM on type B trichothecene accumulation by *Fusarium graminearum* CBS 185.32. Data are means ± standard deviation using three biological replicates. Different letters designate statistically significant differences between data (Kruskal–Wallis one-way analysis, with mean comparisons performed using Connover-Inman test, α ≤ 0.10).

Results on the effect of the seven retained M1 analogs on the TCTB biosynthesis are reported in Figure 4. 

The data showed that analog 2 has a higher inhibitory activity on TCTB production compared to that assessed for the M1 compound. When comparing the chemical structures of M1 and analog 2 (Figure 3), a high similarity between the two compounds clearly appears, the main modification concerning the lack of the CF3 group in M1 replaced by an amide one in analog 2. 

**Figure 5:**
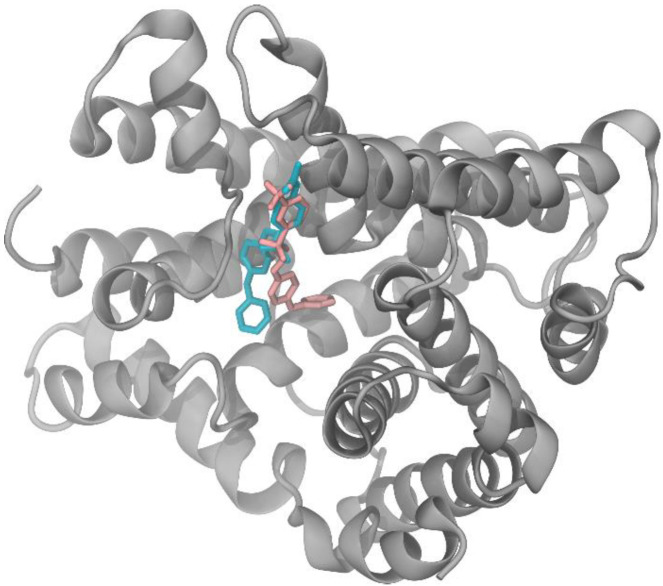
Superposition of M1 (pink) and analog 2 (cyan) docking pauses within the TRI5 protein binding site.

To investigate why analog 2 shows the highest TCTB inhibition activity, a protein/ligand interactions analysis was performed on the best docking pauses of M1 and its seven analogs using both the PLIP [19] and LigPlot+ [20] programs. This allowed us to demonstrate that the docking conformation of analog 2 within the TRI5 protein binding pocket was the one that shows the most similarities with that of M1 (Figure 5).

**Figure 6:**
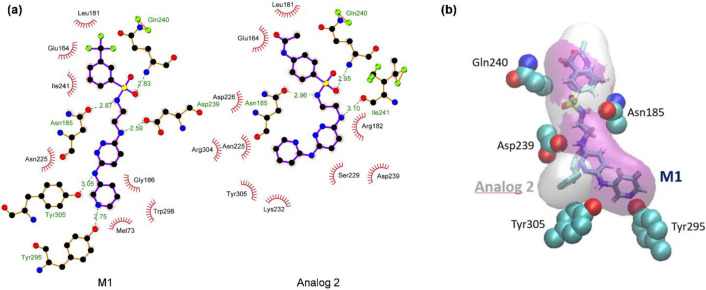
Interaction between TRI5 protein residues with compound M1 and analog 2. (**a**) LigPlot+ picture of the TRI5 in interaction with the compound M1 and analog 2. (**b**) Three dimensional drawing highlighting the main interactions shown in LigPlot picture as ball and stick residues.

As indicated in Table 3, strong molecular interactions were conserved between specific binding pocket residues such as Asn_185_, Asp_239_ and Gln_240_ (Figure 6a,b). Combined with the lower lipophilicity of analog 2 (Table 2), the observations reported above could explain analog 2 experimental behavior compared to that of other compounds.

Four additional M1 analog compounds, namely analogs 3, 4, 5 and 7, were characterized by a higher toxin inhibition effect than M1, while lower than that of analog 2. As indicated in Table 2, analogs 3 and 4 have higher docking scores compared to analog 2, but they both have a slightly higher lipophilicity that could explain their lower effect while presenting a better theoretical affinity. Analog compound 7 is characterized by the lowest lipophilicity but exhibits a poor docking rank. The previous data clearly indicated that, even if docking score must be considered as the most important criterion to predict the compound effectiveness, it is not sufficient to account for all the observed trends. Additional factors such as compound lipophilicity should not be overlooked.

We also checked the effect of the seven retained analogs on the fungus growth. As previously observed for M1, none of these compounds significantly affects the fungal growth when supplemented in the culture media at a 150 µM concentration (data not shown).

## 3. Discussion

The aim of this study was to experimentally validate the bioactivity of drug candidates predicted from a virtual screening campaign to interact with key proteins from the PH1 *F. graminearum* genome involved in the fungal growth and mycotoxin production. While several reports, gathered in the review of Shanmugan and Jeon [12], have attempted to use virtual screening to design new fungicides by selecting targets with a function in fungal development mechanisms, very few of them, if any, have focused on the biosynthesis of mycotoxins. However, since some mycotoxins are acknowledged to play a significant role in fungal virulence, preventing their production will contribute to reduce the disease symptoms. This is the case of DON, production of which has been demonstrated to help *F. graminearum* circumvent plant defense reactions and facilitate its spread within wheat heads [21,22]. Inhibiting specifically the DON production will allow the safety of harvests to be ensured while reducing the fungal disease. Moreover, this strategy should help avoid the unexpected increase in DON production that has sometimes been observed after a fungicide application [23] and is explained by the role played by DON in the adaptive response of *F. graminearum* to stressful conditions [24]. 

According to our results, the M1 top one compound selected for its capacity to interact with a key enzyme of the TCTB biosynthetic pathway was able to considerably reduce the yield of mycotoxins by a set of toxigenic *Fusarium* strains of different chemotypes. This mycotoxin inhibition activity was shown to significantly differ according to the targeted strain, which could be linked to the occurrence of slight variations in the *Tri5* gene nucleotide sequences between the different TCTB-producing strains [25,26], leading to potential modifications of the binding site conformation or to the interaction of the candidate drug with other TRI5 protein partners [27]. Remarkably, at a concentration of 150 µM the M1 top one molecule and several analogs (2, 3, 4, 5 and 7) reduced type B trichothecene production by a factor of up to 70%. Thus, compared to widely studied anti-mycotoxin compounds such as ferulic and caffeic acids that have been shown to be active starting from a concentration of 500 µM [28,29,30], the efficacy of M1 is clearly higher. The in silico characterization of protein/ligand (M1 and analog 2) interactions has allowed several TRI5 amino acid residues with a role in the complexes formation to be highlighted. Among these amino acids, we can mention the Tyr305 residue involved in H-bond with M1 and π -stacking with analog 2. Indeed, Tyr305 that belongs to the “basic motif” of the TRI5 protein is acknowledged for its importance in the binding of farnesyl-pyrophosphate, the precursor of type B trichothecenes [31,32]. Two additional residues, Arg182 and Ser229, also deserve special attention. When Arg182, as Tyr305, is reported as involved in interactions with pyrophosphate through the occurrence of H-bonds, Ser 229 is located in the NSE/DTE motif of TRI5 and is involved in the chelation of one of the three catalytically obligatory Mg^2+^ ions [31]. According to our results, an association with Arg182 and Ser229 is predicted for analog 2, while this is not the case for M1. Combined with the fact that analog 2 has a higher mycotoxin inhibitory capacity compared to M1, the previous observation raises the hypothesis that the occurrence of bonds with Arg182 and Ser229 can importantly contribute to the inhibition of TRI 5 enzymatic activity.

With regard to the top one molecule selected for its high binding affinity to ERG6, a protein involved in the biosynthesis of ergosterol, only a weak reduction in the fungal biomass was observed in our experimental conditions. This weak effect could be explained by the low concentration we used (as a result of the low solubility of the candidate molecule) but also by the non-essential character of ERG6. Indeed, deletion of ERG6 in *Saccharomyces cerevisiae* was shown to impair the membrane fluidity and permeability while not affecting the vegetative growth of the yeast [33]. The non-essential character of ERG6 was also recently corroborated by the study of Konecna et al. [34] who compared the phenotypes of wild type strains of *Kluyveromyces lactis* and of *S. cerevisiae* to those of *ERG6*-deleted strains. For both yeast species, deleted strains were not impacted in their radial growth on YPD plates but were significantly affected in their sensitivity to several growth inhibitors. Thus, it would be relevant to associate molecules interacting with ERG6 with other cell-wall perturbing agents in future research aimed at developing new and efficient plant care products.

Additionally, while confirming the bioactivity of compounds selected through a virtual screening campaign, our study also evidenced that in addition to docking scores and in silico molecular interaction predictions, physiochemical properties of the candidate compounds are likely to significantly contribute to their inhibition efficiency. In particular, when comparing the mycotoxin inhibition efficiency of a set of analog compounds, we suggested that a combination of the two criteria, docking score and lipophilicity, was required to partially explain and predict the bioactivity ranking of the tested molecules. However, while lipophilicity is acknowledged as a key property facilitating the penetration of bioactive molecules through non-polar fungal cell membranes, it is also important to keep in mind that the relationship between lipophilicity and antifungal activity frequently does not follow a linear equation but most often a polynomial one [35]. 

Overall, our data support the promising contribution of computational approaches to identify new pesticides and provide first evidence of their promising use to identify mycotoxin inhibitors. In future research, hypotheses raised by the present study on the importance of ligand interactions with some TRI5 amino acid residues, if proven, can be used as guides to screen natural compound libraries and therefore contribute to the definition of innovative biopesticides to control the contamination of crops with mycotoxins. They will help to identify eco-friendly strategies to control cereal disease and, therefore, answering the critical and growing need for solutions to replace the use of synthetic chemicals, which have potentially deleterious effects on human health and the environment, giving cause for serious concerns.

## 4. Materials and Methods

### 4.1. Fusarium Strains

Eight different *F. graminearum* and *F. culmorum* strains were used in this study: two *F. graminearum* strains with the DON/15-ADON chemotype (PH-1 and CBS 185.32) and six *F. culmorum* strains (three with the DON/3-ADON chemotype-MCf21, Fc 124, and Fc 305- and three with the NIV/FX chemotype-Fc 130, Fc 319 and Fc337). Characteristics of the eight strains are summarized in Table 1. The fungal species’ identity was confirmed using the species-specific primers Fc01 and Fg16N markers [36]. Toxigenic potential of each strain was assessed according to the procedure described by Bakan et al. [37]. 

Stock cultures were maintained at 4 °C on Potato Dextrose Agar (PDA) (Difco, Bordeaux, France) slants under mineral oil. When inoculum was required, the *Fusarium* strains were grown on PDA slants at 25 °C in the dark for 7 days. Spore suspensions were prepared in carboxymethyl cellulose medium as described by Montibus et al. [38] and concentration was determined using a spores counting method.

### 4.2. Medium and Culture Conditions

Liquid culture experiments were performed in a Mycotoxin Synthetic medium (MS medium) [28]. Sterile petri dishes (55 mm in diameter) containing 8 mL of MS medium, supplemented or not with M1 and M2 compounds and analogs, were inoculated with 2 × 10^4^ spores/mL. Fungal liquid cultures (static) were incubated in dark at 25 °C for 11 days. Following incubation, cultures were centrifuged at 3000× *g* for 10 min. Supernatants were stored at −20 °C until TCTB analysis. Fungal biomass was measured by weighing the mycelial pellet after 48 h of freeze-drying (Cryotec, Saint-Gély-du-Fesc, France). According to the solubility of M1 and M2 and to the fungal toxicity of DMSO, the two compounds were tested at 50 and 150 µM for M1, and 5 and 15 µM for M2 in 1% and 3% of DMSO for the first and the second concentrations, respectively. Cultures were made in triplicate. Appropriate controls using compound-free, 1% and 3% DMSO control media and non-inoculated control media were completed. It was verified that the initial pH (pH = 6.5) of the culture medium was not affected by the compound treatment

### 4.3. Extraction and TCTB Quantification

A 4 mL sample of culture medium was extracted with 8 mL of ethyl acetate. Six milliliters of the organic phase were evaporated to dryness at 50 °C under nitrogen flux. Dried samples were dissolved in 200 µL of methanol/water (1/1, *v/v*) and filtered through a 0.2 µm filter before analysis. TCTB were quantified by HPLC-DAD using an Agilent Technologies 1100 series liquid chromatograph equipped with an auto-sampler system, an Agilent photodiode array detector (DAD) and the ChemStation chromatography manager software (Agilent Technologies, Les Ulis, France). Separation was achieved on a Kinetex XB-C18 100 Å column (4.6 × 150 mm, 2.6 μm) (Phenomenex, Le Pecq, France) maintained at 45 °C. The mobile phase consisted of water acidified with ortho-phosphoric acid to pH 2.6 (solvent A) and acetonitrile (solvent B). The flow was kept at 1 mL min^−1^. The injection volume was set to 5 μL. TCTB were separated using a gradient elution as follows: 7 to 30% B in 10 min, 30–90% B in 5 min, 90% B for 5 min, 90 to 7% B for 2 min, 7% B for 5 min. The UV–Vis spectra were recorded from 190 to 400 nm and peak areas were measured at 230 nm. Quantification was performed by external calibration with standard solutions (Romer Labs, Baulkham Hills, Austria).

### 4.4. Expression of Results and Statistical Analyses

Results for fungal biomass and TCTB production were reported as mean values ± standard deviation of three biological replications.

Since the data were non-normally distributed (Shapiro–Wilk normality test), the comparison of values (control vs. treated) was carried out with Mann–Whitney test (unpaired test). To compare the score of more than two groups, Kruskal–Wallis one-way analysis was used, with mean comparisons performed using Connover-Inman test (*p* ≤ 0.10). Statistical analyses were conducted with XLSTAT 2017 software (Addinsoft, Rennes, France).

### 4.5. Molecular Docking Analysis

As described in Martins et al. [13], the GOLD software was used for performing the molecular docking using the same conditions and the same target structures. 

The chemical library used was the one providing a high diversity of compounds and was accessed from Life Chemicals [https://lifechemicals.com/ (accessed on 10 October 2019)]. These libraries contained initially 50 K compounds, but after clustering them in order to retain only the most different scaffolds, 10,240 molecules were retained and each one was docked within the binding site of the selected targets. 

As several stable conformers were identified for each TRI5 and ERG6 targets, we used the ensemble docking possibility available in GOLD. The use of such conformational ensembles was considered as an improved strategy in structure-based docking calculations. Prior to the docking itself, the target conformers used were aligned in a common reference system and the center of the binding pocket cavity used by the ensemble docking procedure was an average of the individual centers found in each conformation. A sphere of 15 Å was selected to define the binding region around this center.

For each ligand docking, 100 starting ligand conformers were considered in GOLD searches for obtaining the best poses. GOLD default parameters with CHEMPLP scoring function were used.

## Figures and Tables

**Table 1:** Effect of 150 µM M1 on type B trichothecene production by eight *Fusarium* strains.

Strain	Chemotype	Source	Sum of Type B Trichothecenes (µg g^−1^ of Dry Biomass)	
			Control	M1
*F. graminearum*				
CBS 185.32	DON/15-ADON	CBS Collection, The Netherlands	38,379.2 ± 1059.8	24,391 ± 1314.2 *
PH-1	DON/15-ADON	The Fungal Genetics Stock Center, USA	76.8 ± 13.3	<LOQ *
*F. culmorum*				
Fc 130	NIV/FX	INRAE, MycSA, France	157.8 ± 7.6	107.3± 8.3 *
Fc 337	NIV/FX	INRAE, MycSA, France	166.1 ± 18.5	126.2 ± 5.5 *
Fc 319	NIV/FX	INRAE, MycSA, France	7617.3 ± 1026.6	6565.7 ± 483.6
MCf 21	DON/3-ADON	University of Sassari, Italy [16]	185.5 ± 68.6	148.4 ± 16.8
Fc 124	DON/3-ADON	INRAE, MycSA, France	304.9 ± 29.3	148.7 ± 24.2 *
Fc 305	DON/3-ADON	INRAE, MycSA, France	5994.1 ± 39.1	6465.7 ± 547.7

* Significant difference with control treatment (Mann–Whitney test, α ≤ 0.10). Data are means ± standard deviation using three biological replicates. LOQ, limit of quantification (30 µg g^−1^).

**Table 2:** The M1 analogs retained for experimental tests.

Compound	Label	Chemical Formula	GOLD Score	GOLD Rank	Estimated Lipophilicity (LogP)
F5258-0045	M1	C_18_H_17_F_3_N_6_O_2_S	80.5	1	4.30
F5238-0129	6	C_19_H_20_N_6_O_4_S	74.1	3	3.13
F5238-0094	3	C_18_H_20_N_6_O_2_S	73.9	4	3.39
F5233-0112	1	C_20_H_23_N_7_O_3_S	73.3	7	2.72
F5238-0098	4	C_18_H_20_N_6_O_3_S	71.9	9	3.16
F5235-0111	2	C_19_H_21_N_7_O_3_S	69.8	19	2.22
F5238-0101	5	C_19_H_22_N_6_O_4_S	68.4	27	2.86
F6363-0589	7	C_19_H_23_N_5_O_5_S	67.4	34	1.95

**Table 3:** Consensus PLIP/LigPlot + protein/ligand interactions between M1 and analog 2.

Residue N°	Type of Interaction	M1	Analog 2
Glu_164_	polar	+	+
Leu_181_	hydrophobic	+	+
Arg_182_	H-bond	−	+
Asn_185_	H-bond	+	+
Asn_225_	H-bond	+	+
Ser_229_	H-bond	−	+
Asp_239_	H-bond polar	+ −	− +
Gln_240_	H-bond	+	+
Ile_241_	H-bond hydrophobic	+ +	+ +
Tyr_295_	H-bond	+	−
Trp_298_	π stacking	+	−
Tyr_305_	H-bond π stacking	+ −	− +

+ means that the interaction exists with the residue; − means that interaction was not found.

## Data Availability

Research data are available from the authors.

## Sample Availability

Samples of the compounds are available from the authors. INRAE-MycSA strains are deposited in the International Center for Microbial Resources-Filamentous Fungi (https://www6.inra.fr/cirm_eng/Filamentous-Fungi (accessed on June 2013)).
